# Cone-beam computed tomography-guided volumetric assessment of secondary alveolar bone grafting

**DOI:** 10.1038/s41598-025-15083-9

**Published:** 2025-08-13

**Authors:** Julian A. Erkert, Benito K. Benitez, Yoriko Lill, Sebastian Tapia Coron, Andreas A. Mueller

**Affiliations:** 1 Oral and Craniomaxillofacial Surgery, University Hospital Basel and University of Basel, Spitalstrasse 21, Basel, 4031 Switzerland; 2 Facial and Cranial Anomalies Research Group, Department of Clinical Research, Department of Biomedical Engineering, University of Basel, Basel, Switzerland; 3 Pediatric Oral and Craniomaxillofacial Surgery, University Children’s Hospital Basel, Spitalstrasse 33, Basel, Switzerland

**Keywords:** Cone-Beam computed tomography, Alveolar bone grafting, Cleft lip, Cleft palate, Three-Dimensional imaging, Bone imaging, Radiography, Paediatric research

## Abstract

This study aimed to develop and implement a standardized 3D workflow for surgical planning and outcome assessment in secondary alveolar bone grafting (SABG) using cone-beam computed tomography (CBCT). A 3D protocol was developed using medically certified software for semiautomatic segmentation, mirroring, voxel-based alignement of the maxilla and volumetric analysis of cleft and graft regions. This workflow was applied on a retrospective cohort of patients with unilateral cleft lip and alveolus (CLA) and/or cleft lip and palate (CLP) who received iliac bone mixed with synthetic biphasic calcium phosphate for bone grafting. CBCT scans (preoperative, immediate postoperative, and six-months follow-up) from 23 patients with unilateral clefts were analysed. The non-cleft side was mirrored to serve as reference for symmetric reconstruction. Measured parameters included alveolar cleft volume, grafted bone volume, integrated bone volume and missing volume. The workflow enabled reliable volumetric quantification. The preoperative cleft volume averaged 1505 mm^3^ (SD 425 mm^3^) and graft volume 1983 mm^3^ (SD 407 mm^3^). After six months, the mean integrated bone volume was 851 mm^3^ (SD 294 mm^3^), corresponding to a mean graft resorption of 57% (SD 13%) and alveolar cleft restoration of 58% (SD 19%). This 3D CBCT-based protocol provides a robust methodological framework for assessing SABG outcomes. It enhances quantification of cleft morphology and graft integration, supporting improved surgical planning, follow-up, and cross-study comparability.

**Supplementary Information:**

The online version contains supplementary material available at 10.1038/s41598-025-15083-9.

## Introduction

In patients with orofacial clefts involving the alveolus, alveolar bone grafting is performed to restore the continuity of the maxillary alveolar process. Adequate bone stock in the former cleft region is a prerequisite for eruption of permanent teeth and subsequent orthodontic treatment^1^. Moreover, it contributes to improved aesthetic outcomes by providing support for harmonious and symmetrical lip and nasal soft tissues^1,2^. Secondary alveolar bone grafting (SABG) is commonly performed secondary to the primary repair of cleft lip and palate, with the gold standard being the use of autogenous bone grafts from the iliac crest.

The volume of integrated bone, also commonly referred to as residual bone graft volume, is a key indicator of SABG success, as it is prone to resorption^3^. Accurate and reproducible measurement of this volume is therefore essential for evaluating surgical outcome. Traditionally graft assessment has relied on two-dimensional (2-D) imaging methods using panoramic, periapical, and occlusal radiographs^4^. Abyholm et al.^5^ first introduced a radiographic grading system in 1981. This system was later established as the Bergland Scale, now widely used to assess alveolar bone height^2,6^. Other scales, like the Chelsea Scale^6^, which evaluates vertical and horizontal bone formation, as well as modifications like the scale by Hynes et al.’s^7^ for basal bone evaluation, have since been developed.

While 2-D radiographs are low-cost and low-radiation, they are limited by image distortion, superimposition and lack of spatial reference points, factors that compromise accurate estimation of the cleft’s true 3-D anatomy^8–10^.

**Fig. 1:**
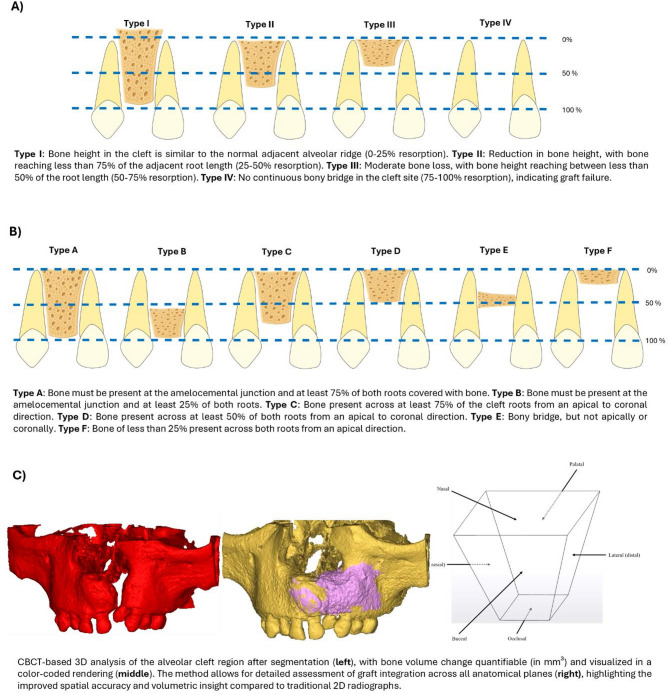
Comparison of 2D and 3D imaging modalities for the assessment of alveolar bone regeneration following secondary alveolar bone grafting. (**A**) **Bergland classification**, based on periapical radiographic assessment which grades alveolar bone height in relation to adjacent teeth from complete bone fill (Type I) to absence of bone bridge (Type IV). (**B**) **Chelsea classification**, based on periapical radiographic evaluation which quantifies bone fill based on the percentage of root coverage both vertically and horizontally. (**C**) **3D volumetric assessment**, CBCT-based 3D analysis following segmentation of the cleft region, with alveolar cleft bone volume change visualized in a color-coded model. This approach allows for assessment in all planes providing improved anatomical accuracy and volumetric quantification compared to 2D imaging.

3D imaging techniques, such as cone-beam computed tomography (CBCT), have been shown to overcome these limitations by providing more accurate assessments of SABG outcomes^8,11–13^. Preoperative estimation of the required graft material through 3-D imaging allows for precise planning, which can improve bone graft stability and reduce donor-site morbidity^14–16^. Furthermore, 3-D assessments enable structural and volumetric evaluation of surgical techniques and materials, contributing to the refinement of the complex therapy. The most used methods for assessing SABG success involve volumetric measurements of the bone graft volume and bone resorption rates^17^. Postoperative assessment is possible by obtaining additional datasets after bone grafting^18^. Frequently used 3-D imaging modalities include computed tomography (CT) and multi-slice CT, but cone-beam computed tomography (CBCT) is also used for volumetric assessment of cleft palate^17^ to lower radiation exposure aligning with the “As low as reasonably achievable (ALARA)” principle^19,20^. Figure 1 compares 2D and 3D radiographic assessment methods for postoperative evaluation of alveolar bone grafting, highlighting the improved volumetric accuracy offered by CBCT analysis.

Standardized outcome criteria and consensus on optimal evaluation timing remain lacking^17^. Additionally, 3-D image analysis is observer dependent, limiting reproducibility and generalizability^21^. Although recent studies have proposed methodologies to address this^22–25^, a comprehensive, user-friendly workflow that promotes standardization without requiring advanced computer skills is still missing.

This study aimed to establish a clinically applicable, standardized 3D assessment workflow for evaluating alveolar clefts using CBCT data. The primary objective was to develop a comprehensive workflow that consolidates key methodological components into a single protocol using available commercial software. As a secondary objective, our workflow was applied to datasets of patients presenting with unilateral cleft lip and alveolus (CLA) and/or cleft lip and palate (CLP) to evaluate its feasibility and potential clinical utility.

## Materials and methods

**Fig. 2:**
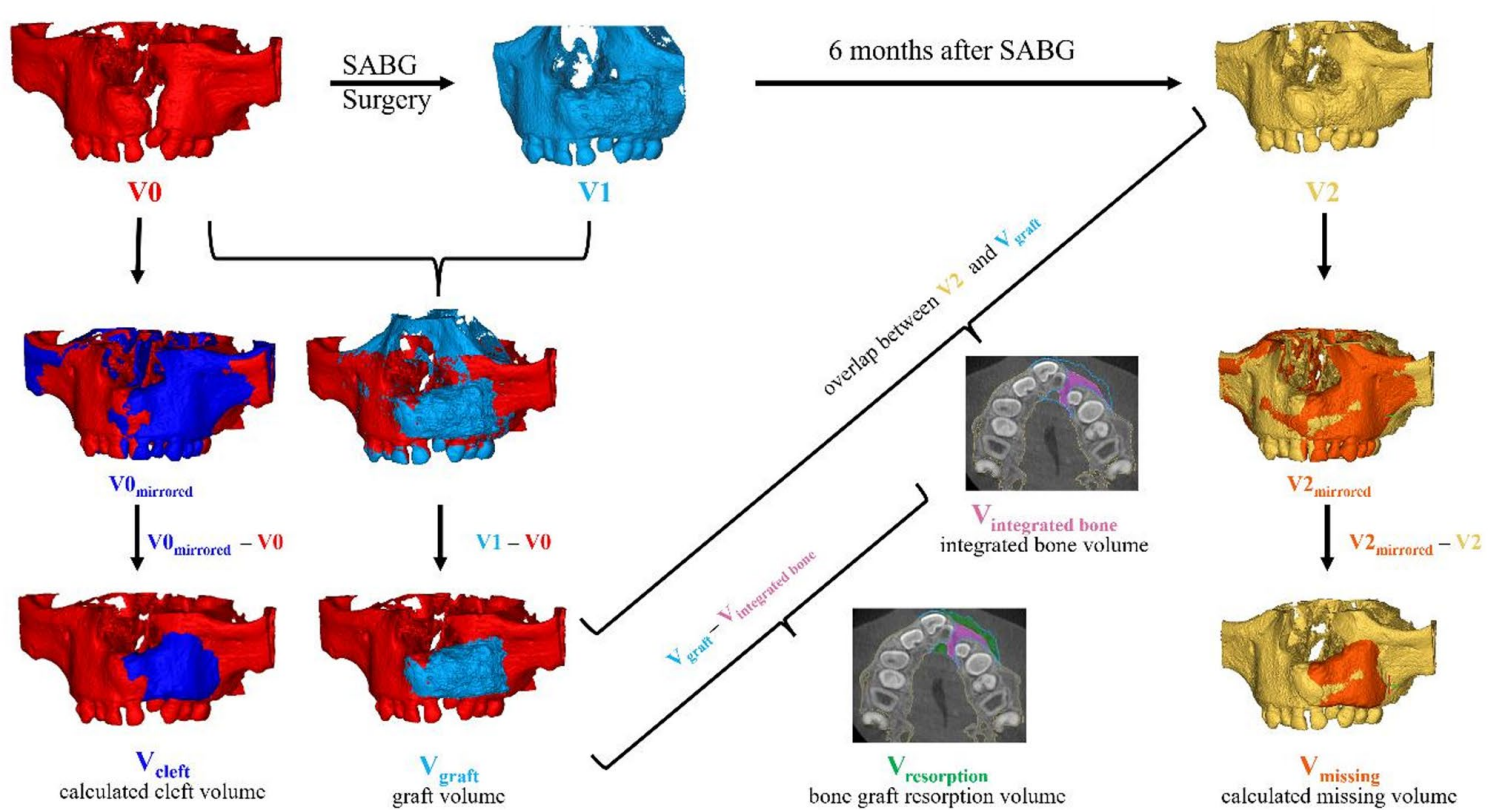
Overview of the workflow starting with DICOM files and segmentation of the dataset before surgery (**V0**) to calculate the cleft volume (**V**_**cleft**_), after secondary alveolar bone grafting (SABG) (**V1**) to quantify the graft volume (**V**_**graft**_) and after 6 months follow-up (**V2**) for subsequent volumetric analysis of integrated bone volume (**V**_**integrated bone**_), bone graft resorption volume (**V**_**resorption**_) and calculated missing volume (**V**_**missing**_).

We developed a standardized, 3D software-assisted workflow for surgical planning and follow-up evaluation of alveolar bone grafting using pre- and postoperative CBCT-Scans in children with unilateral cleft lip and alveolus (CLA) and/or cleft lip and palate (CLP). An overview of the workflow is shown in Fig. 2 and Supplemental Video 1. To evaluate its feasibility and clinical applicability, the workflow was retrospectively applied to a cohort of patients who received secondary alveolar bone grafting with a mixture of autologous iliac crest bone and synthetic biphasic calcium phosphate.

### Workflow development process

The workflow was developed using Materialise Innovation Suite (version 25.0, Materialise Nv, Leuven, Belgium). Image datasets were in Digital Imaging and Communication in Medicine (DICOM) format. All the steps involved in the analytical protocol consisting of segmentations and volumetric analyses were established in close consultation with two experienced surgeons, to ensure methodological accuracy and clinical relevance of the volumetric measurements. A protocol describing in detail the workflow using Materialise Suite is available in the Open Science Framework (Link). The workflow can easily be applied to other 3D data software that are equipped with features used in the presented protocol.

### Segmentation

**Fig. 3:**
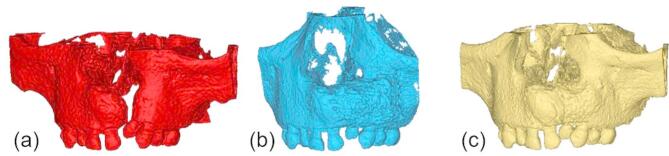
Three-dimensional models of the maxilla with alveolar cleft on the left side after segmentation and calculation of a standard triangulated language-file: (**a**) preoperatively **V0** (red), (**b**) immediately after early secondary alveolar bone grafting **V1** (blue) and (**c**) 6 months after surgery **V2** (yellow).

The initial step in the workflow is to align the DICOM datasets acquired at three time points; before SABG surgery, immediately after surgery, and at 6 months follow-up by re-slicing the images parallel to the occlusal plane to correct for potential variations in head positioning during image acquisition. Next, the teeth and bones are segmented separately, each using specific threshold settings. After merging both segmentation masks, gaps are filled to cover the entire cortical bone and teeth. The cancellous bone is filled semi-automatically to obtain a dense model. Figure 3 presents 3D reconstructions (preoperative **V0**, immediate postoperative **V1**, and six-months follow-up **V2**) exported in standard triangulated language (STL) format following segmentation and subsequently imported into Materialise 3-matic (Version 15.0, Materialise Nv, Leuven, Belgium) for further processing.

### Volumetric analysis

The following volumes are calculated based on the segmented models obtained from the V0, V1, and V2 datasets.

1. Calculated Cleft Volume based on contralateral reference (**V**_**cleft**_ = **V0**_**mirrored**_ - **V0**).
2. Graft Volume (**V**_**graft**_ = **V1** - **V0**).
3. Integrated Bone Volume (**V**_**integrated bone**_= overlap of **V2** and **V**_**graft**_ = **V2** $\:\cap\:\:$**V**_**graft**_).
4. Bone Graft Resorption Volume (**V**_**resorption**_ = **V**_**integrated bone**_– **V**_**graft**_).
5. Volumetric deficit estimate based on contralateral reference (**V**_**missing**_ = **V2**_**mirrored**_ – **V2**).

The following sections describe the volumetric calculations in detail.

1. Calculated Cleft Volume (**V**_**cleft**_) based on contralateral reference **(V0**_**mirrored**_ **- V0 = V**_**cleft**_**)**.

**Fig. 4:**
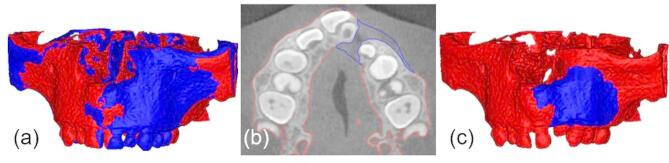
Superimposition of V0 (red) and V0_mirrored_ (dark blue) before manual post-processing (**a**) and after manual post-processing in axial view (**b**) and in 3D view (**c**).

The unaffected side is used as the reference to calculate the initial cleft volume (**V**_**cleft**_). A duplicate of the original V0-model is mirrored along the median palatine suture, perpendicular to the maxilla, only to define the initial spatial position of the mirrored anatomy. Precise alignment is then achieved through voxel-based registration, which maximizes 3D overlap between mirrored and original model. Figure 4**(a)** shows the superposition of **V0** model and mirrored **V0** model after manual alignment and subsequent automatic voxel-based registration. The same alignment procedure is used for all further superimpositions. The **V0** model is subtracted from the **V0**_**mirrored**_ model to calculate the initial cleft volume (**V**_**cleft**_). The model obtained by subtraction is trimmed according to anatomical landmarks, and contours around the transition of the cleft volume to the **V0** model are smoothened to account for asymmetries surrounding the cleft (Fig. 4**(b) and (c)**).

2. Graft Volume (**V**_**graft**_) **(V1 - V0 = V**_**graft**_**)**.

**Fig. 5:**
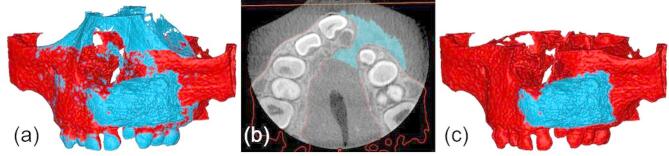
Superimposition of V0 (red) and V1 (blue) (**a**). V_graft_ after subtraction and manual post-processing in axial view (**b**) and in 3D view (**c**).

Due to the low contrast in CBCT scans, the graft volume cannot be clearly delineated from the former cleft defect. Therefore, the **V0** and **V1** models are overlaid to define the bone graft boundaries (Fig. 5**(a)**). The graft volume (**V**_**graft**_) is obtained by subtracting the **V0** model from the **V1** model, followed by manual post-processing (Fig. 5**(c)**). Contours are controlled using 2D images in Mimics software (Fig. 5**(b)**).

3. Integrated Bone Volume (**V**_**integrated bone**_) and Bone Graft Resorption Volume (**V**_**resorption**_).

**(Overlap between V2 and V**_**graft**_ **= V2** $\:\cap\:\:$ **V**_**graft**_ **= V**_**integrated bone**_**)** and **(V**_**integrated bone**_**– V**_**graft**_ **= V**_**resorption**_**)**.

**Fig. 6:**
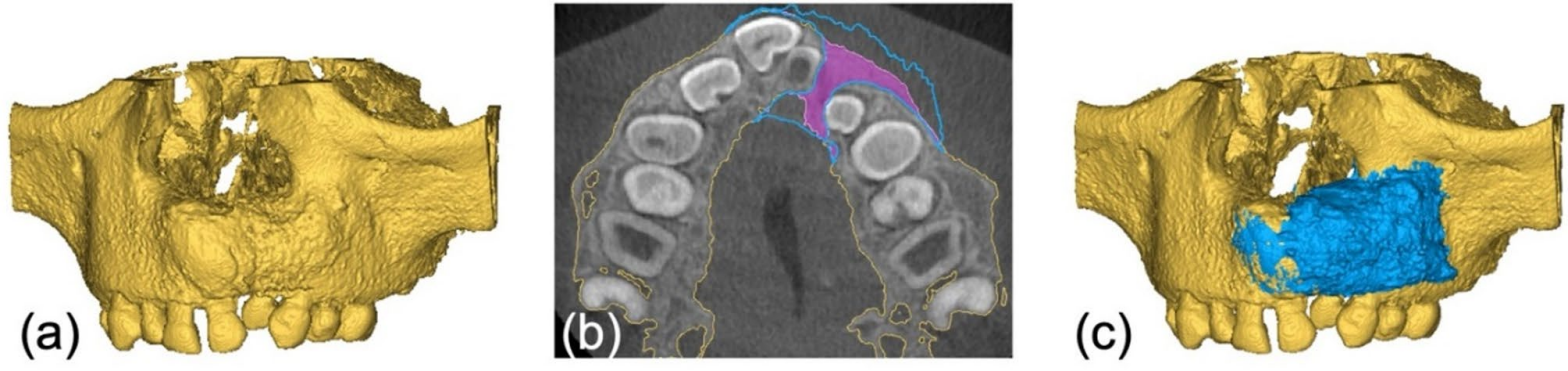
Model V2 from 6 months postoperative CBCT (**a**), V_graft_ (blue) superimposed on V2 (yellow), and V_integrated bone_ (pink) which is the overlap between V2 (yellow) and V_graft_ in axial view (**b**), V_graft_ superimposed on V2 (yellow) in 3D view (**c**).

After six months of healing, the boundary between the original bone graft and the surrounding native maxillary bone typically becomes indistinct on CBCT imaging. To quantify the extent of successful graft integration, the integrated bone graft (**V**_**integrated bone**_) is defined as the overlapping volume between the initial graft volume **V**_**graft**_ and the segmented bone volume at six months postoperatively (**V2)** (Fig. 6**(a-c)**). In this context, **V**_**integrated bone**_ represents the portion of the original graft that remains occupied by mineralized tissue within **V2.** The resorbed bone graft volume, **V**_**resorption**_, is then calculated as the difference between the original graft volume and the integrated portion:

**V**_**resorption**_ = **V**_**graft**_ - **V**_**integrated bone**_.

This method ensures that only the mineralized tissue that originated within the spatial boundaries of the initial graft is counted as integrated, despite the absence of visible borders on follow-up imaging.

4. Volumetric deficit (**V**_**missing**_) estimate based on contralateral reference **(V2**_**mirrored**_ **– V2 = V**_**missing**_ **)**.

**Fig. 7:**
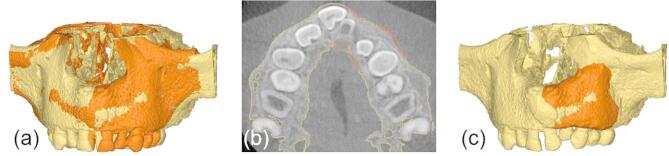
Superimposition of V2 (yellow) and V2_mirrored_ (orange) before manual post-processing **(a)**, and after manual post-processing in axial view (**b**) and in 3D view (**c**) to estimate volumetric deficit (**V**_**missing**_) relative to contralateral reference.

As bone resorption is greater when the cleft is overfilled, the resorption rate alone is not a reliable outcome parameter. The volume necessary to achieve the optimal contour of the maxillary arch (**V**_**missing**_) is defined as an additional endpoint and approximated by mirroring the healthy side (Fig. 7**(a-c)**). The **V2**_**mirrored**_ model is subtracted from **V2** to obtain the missing volume (**V**_**missing**_) for symmetry.

### Original clinical data

Included were CBCT scans of patients with non-syndromic unilateral cleft lip and palate or unilateral cleft lip and alveolus undergoing early SABG before eruption of the permanent incisor adjacent to the alveolar cleft. Informed consent was obtained from the parents or guardians of the children. The study was performed in accordance with the Declaration of Helsinki and was approved by the Ethics Commission (Project ID, 2019-00226). CBCT scans were acquired preoperatively, one week postoperatively, and 6 months postoperatively using a 3D Accuitomo 170 Imaging System (J. Morita Manufacturing Corp. Kyoto, Japan). The scans were performed at 80–90 kV and in 6 mA pulse mode, using a voxel size ranging from 0.125 to 0.160 mm. The field of view (FOV) ranged from 6.125 × 6.125 × 6.5 cm to 8.16 × 8.16 × 8.640 cm. The exposure time was 9.0 s. All patients had a natural head position during the scanning. All operations were performed at a cleft centre by the same senior cleft surgeon between January 2014 and December 2020. The surgical technique was standardized and performed in all the included patients as follows: The alveolar cleft was exposed to the entire vertical, transverse, and buccolingual extent. In case of oronasal fistula, the palatal mucosa and the nasal layer was sutured until a completely tight seal of the nasal floor was present, from palatal to buccal, across the alveolar cleft. Tightness was tested using a bulb-headed probe. Cortico-cancellous iliac bone was harvested from the inner cortex, crushed and mixed with synthetic biphasic calcium phosphate (OSOPIA, Regedent AG Zürich, Switzerland) containing > 90% ß-tricalcium phosphate (ß-TCP) and < 10% hydroxyapatite (HA). The ratio of the substitute material was limited to an estimated 1⁄4 to 1⁄3 of the total graft volume. The cleft was filled in all dimensions including the bone deficit around the alar base and nasal floor, and sagittal deficit on the buccal cleft side. After compression, the bone graft was covered buccally with a decellularized freeze-dried Amnion-Chorion membrane (ACMTRIX, TBF, Mions, France). Wound closure was performed with papilla sutures without tension.

### Statistical analysis

Descriptive statistical analysis to summarise the measurements was performed using Python^®^ (Python Software Foundation. Python Language Reference version 3.9.13). Sample size was limited by the number of consecutive cases with consistent surgical procedure as well as fulfilling the inclusion criteria.

## Results

### Workflow development and implementation

We developed an objective workflow for volumetric assessment of alveolar clefts and graft using CBCT data obtained in clinical routine and medically certified 3D imaging software for the data processing. The protocol is based on segmentation of CBCT DICOM data and includes a series of standardized steps in 3D data manipulation; image registration, mirroring of the non-cleft side, and application of Boolean operations on the segmented 3D meshes. This approach allows for precise definition and measurement of cleft volume before surgery for planning, graft volume immediately after the surgery, and integrated volume at a follow-up for outcome assessment.

### Application to patient cohort

**Fig. 8:**
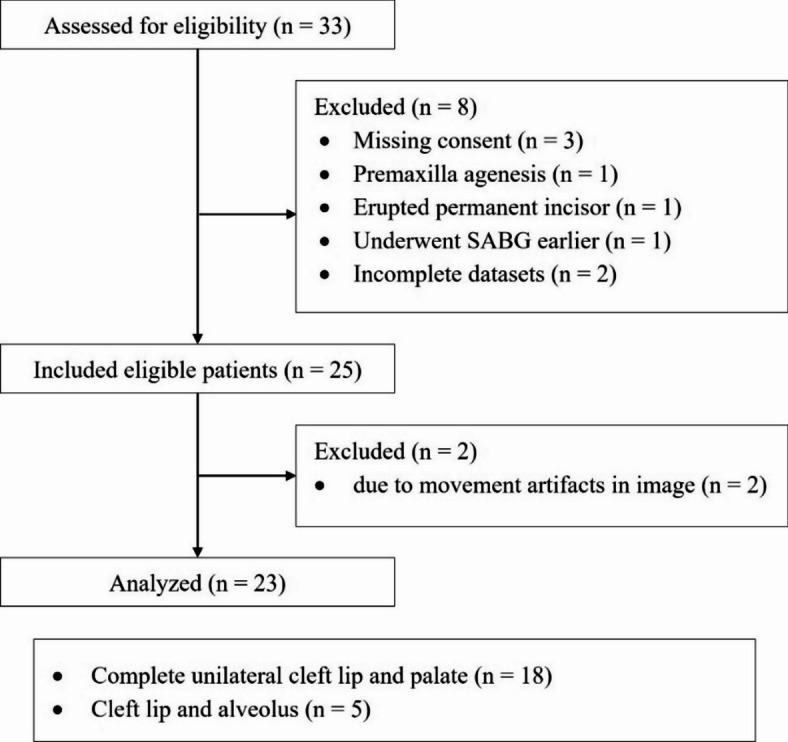
Flow diagram of patient inclusion.

The workflow was applied retrospectively to a cohort of 23 patients who underwent alveolar bone grafting. The median age at surgery was 5.9 years (interquartile range (IQR) = 5.7–6.2 years). The selection process is summarized in Fig. 8 and baseline characteristics of the included patients are presented in Table 1.

**Table 1:** Distribution of the baseline characteristics of the patients included in the study.

	Total (*n*)	CLA (right / left)	CLP (right / left)
**Total (n)**	23	5 (0 / 5)	18 (8 / 10)
**Male (n)**	18	3 (0 / 3)	15 (7 / 8)
**Female (n)**	5	2 (0 / 2)	3 (1 / 2)
**Age at surgery in months (mean [sd])**	6.3 [1.3]	6.9 [1.5]	6.1 [1.2]

For each case, the following volumes were measured:

- Initial cleft volume (pre-operative).
- Graft volume (immediately post-operative).
- Integrated bone volume (6 months post-operative).
- Volumetric deficit estimate based on contralateral reference (6 months post-operative).

Furthermore, based on the volumes, clinically relevant parameters were calculated:

- Filling Rate.
- Alveolar Cleft Restoration.
- Bone Graft Resorption.

### Quantitative outcomes

Quantitative variables determined in the study to test the developed workflow in this study are shown in Table 2.

**Table 2:** Mean and standard deviation (SD) of the calculated volumes, and filling rate, alveolar cleft restoration, and bone graft resorption.

	Description	Mean (SD)
**V**_**cleft**_ **(mm**^**3**^**)**	Calculated cleft volume for symmetry	1505.2 (425.3)
**V**_**graft**_ **(mm**^**3**^**)**	Grafted volume after surgery	1982.7 (406.6)
**V**_**integrated bone**_ **(mm** ^**3**^ **)**	Integrated bone volume six months after surgery	850.8 (293.8)
**V**_**missing**_ **(mm**^**3**^**)**	Calculated missing volume based on contralateral reference	867.6 (380.1)
**Filling Rate (%)**	Ratio of the graft volume (V_graft_) to the calculated cleft volume (V_cleft_)	136.3 (29.9)
**Alveolar Cleft Restoration (%)**	Proportion of V_cleft_ that has been reconstructed with V_integrated bone_	57.9 (18.5)
**Bone Graft Resorption (%)**	Comparing initial graft volume with integrated bone volume	56.6 (13.0)

**Fig. 9:**
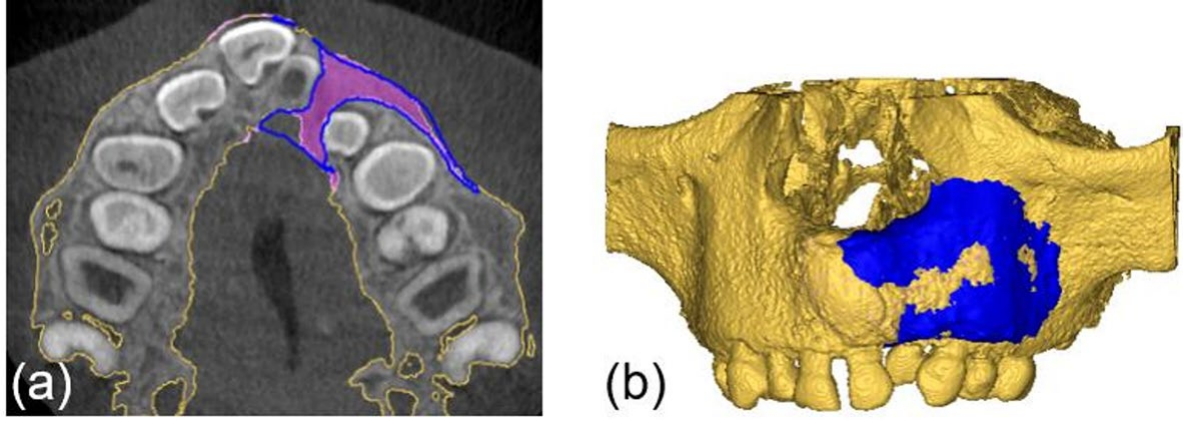
Superimposition of V2 (yellow, 6 months postoperative) with V_integrated bone_ (pink) and V_cleft_ (dark blue) in axial view (**a**) and in 3-D view (**b**), illustrating the proportion of the cleft volume that has been filled with integrated bone. This proportion represents the alveolar cleft restoration rate.

Alveolar cleft restoration (%) quantifies the extent to which the initial cleft defect has been reconstructed with integrated bone tissue. It is calculated by comparing the volume of integrated bone 6 months after surgery to the cleft volume required to achieve symmetrical maxillary morphology. Based on this approach, as shown in Fig. 9, an average alveolar cleft restoration of 58% (SD 19%) was achieved. On average, 868 mm^3^ (SD 380 mm^3^) was missing on the cleft side to achieve perfect symmetry based on the symmetry analysis by mirroring the segmented maxillary bone six months after SABG.

The bone graft resorption rate is determined by comparing the initial bone graft volume with the integrated volume after six months. The difference between the original graft volume and the integrated volume represents the amount of graft resorption, which is expressed as a percentage of the original volume, with a mean of 57% (SD 13%).

The filling rate defined as the ratio of the graft volume to the calculated cleft volume required for symmetrical reconstruction was lower for larger cleft volumes (correlation=-0.55, p-value = 0.01); nevertheless, overfilling of the cleft did not result in a higher bone graft resorption rate (correlation = 0.33, p-value = 0.13) nor in a higher alveolar cleft restoration rate (correlation = 0.41, p-value = 0.05).

## Discussion

Integrated bone volume is a key parameter in determining the success of alveolar bone grafting and has been used to compare outcomes across different graft materials^22^. Accurate determination of pre-surgical cleft volume is clinically important for surgical planning; both to optimize graft quantity and to predict outcomes. However, it remains challenging due to poorly defined anatomical landmarks within the cleft defect and the lack of a standardized assessment method^17,21^. The primary objective of this study was to develop a standardized 3-D assessment workflow using CBCT, in which the bone of the unaffected side of the maxilla is mirrored using specialized 3-D software to objectively evaluate symmetric bony reconstruction in cleft malformations^23,24,26^.

The workflow, which combines segmentation, registration, mirroring and Boolean operations, enable standardized quantification of cleft volume before surgery, graft volume immediately after surgery, and integrated and missing bone volume at follow-up^22–25^. A major advantage of this workflow is its ability to minimize subjectivity in assessing the alveolar cleft and graft integration. By leveraging 3D image analysis and consistent segmentation protocols, our method has the potential to provide a reliable framework for preoperative planning and postoperative monitoring. Traditional 2D assessment lacks the spatial precision required for surgical planning and outcome evaluation. Conventional grading systems such as the Bergland and Chelsea scales have long been used to assess alveolar bone graft outcomes. While useful, these 2D classifications rely on radiographs that may not accurately reflect the true three-dimensional anatomy of the cleft region, especially due to limitations such as image superimposition, distortion, and lack of depth perception. In contrast, a 3D workflow, based on CBCT and segmentation techniques, offers a more detailed and quantitative approach. Rather than assigning a qualitative grade based on vertical or horizontal bone fill in a single plane, this method calculates actual volumes, including the preoperative cleft size, graft volume, and the extent of integrated bone over time. In this context, volumetric 3D assessment functions not only as a complementary method but also as a potentially more reliable alternative, particularly in clinical scenarios that demand precision in surgical planning and outcome evaluation.

The workflow can be implemented using widely available software, making it accessible to both clinical and research settings. Furthermore, the protocol’s modular structure allows for adaptation to different grafting techniques or follow-up intervals.

The second objective of this study was to apply the workflow to quantify the volume stability of iliac crest bone grafts enhanced with synthetic biphasic calcium phosphate in patients with unilateral CLA and/or CLP.

The application of the workflow in a cohort of 23 patients demonstrated its feasibility and clinical application. Preoperative cleft volumes averaged 1505.2 mm^3^ (SD 425.3 mm^3^), which were larger than most values reported^23,24,27^ in previous studies due to the inclusion of the buccal defect to achieve maximal symmetry. The alveolar cleft restoration rate, calculated from this preoperative cleft volume, was 57.9% (SD 18.5%). Excluding the two patients where SABG was clinically unsuccessful, alveolar cleft restoration rate would have been 61.4% (SD 4.9%). Ths is consistent with studies using similar mirroring techniques, such as Janssen et al., who reported 61.6% (SD 23%), and Liu et al., who found a rate of 47.7% (SD 16.4%), despite the differences in the definition of preoperative cleft volume and integrated graft volume^23,24,27^. A standardized protocol across studies is necessary for an accurate comparison^17,23,24^.

Average bone graft resorption ratio was 56.6% (SD 13.0%). This is comparable to other studies that measured the graft volume immediately after surgery. For instance, Nagashima et al. reported a mean bone graft resorption ratio of 47.2% (SD 25.9%) six months postoperatively in patients with unilateral CLA and/or CLP^27^, while Feng et al. found an average bone graft resorption of 30.07% (SD 15.73%) one year postoperatively in patients with UCLA who underwent SABG with iliac cancellous bone^18^. The variability in resorption rates highlights the influence of local biological factors on graft integration, indicating the importance of evaluating both volumetric changes and dynamic integration of the graft within the bony cleft^21^.

Some studies have suggested that overfilling the cleft with graft material may lead to a higher resorption rate^28,29^. However, in the present analysis no significant correlation between overfilling and resorption was detected. Overfilling tended to be lower in cases with larger cleft volumes (correlation=-0.55, p-value = 0.01), suggesting that the effect of overfilling on resorption may be masked by the size of the preoperative cleft volume, like the findings of Nagashima et al.^27^. Although previous studies have shown that newly formed bone can be delineated on CT-scans after six months^30^, other evidence indicates that the bone continues to change between six and 12 months postoperatively. Thus, autogenous bone grafts may not have been fully integrated within the timeframe of the present study^17^. Future studies should consider extending the follow-up periods to better understand the long-term implications of SABG. However, due to facial growth and tooth eruption, superimposition of CBCT analysis may become less accurate over longer follow-up periods^31^.

## Conclusion

Preoperative planning and postoperative evaluation of alveolar bone grafting using 3-D-imaging datasets are widely recognized as superior to traditional 2-D assessment techniques. However, challenges remain with CBCT datasets, particularly the low contrast, which complicates the accurate differentiation among tissues. Advances in CBCT technology combined with post-processing techniques may help overcome these limitations. Additionally, ultra-low dose scanning protocols^32^ can reduce radiation exposure while maintaining a sufficient image contrast for precise segmentation.

The workflow presented in this study can be an alternative to traditional 2-D methods for assessing outcomes in children undergoing SABG surgery. Outcome comparisons regarding volume stability across different grafting techniques are only feasible with the accuracy provided by 3-D data. Incorporating AI-assisted automated segmentation tools to replace manual steps in the proposed protocol may further enhance efficiency and reproducibility. Further studies using larger datasets are warranted to validate the approach and strengthen its generalizability. Such validation is a prerequisite for advancing automation of the workflow, consequently facilitating clinical translation.

## Supplementary Information

Below is the link to the electronic supplementary material.

Supplementary Material 1

## Data Availability

The datasets used and/or analysed during the current study are available under Opens Science Framework along with our Standard Operating Procedure of the protocol. https://osf.io/vkwg5/files/osfstorage?view_only=61ffd405078543d2a98ef8187e351355.
